# Treatment of Malignant Disease

**Published:** 1844-03-09

**Authors:** 


### TREATMENT OF MALIGNANT DISEASE.

The general rules for the treatment of malignant disease are these:—If the case is decided, any palpable disorder in the health should first be removed, by alteratives and tonics, and then the disease be extirpated as soon as possible, provided it can be done with safety. If the case is doubtful, an alterative plan of treatment must be pursued, which will cure the disease if it be not really malignant,— retard its progress if it be. Again, if the case is decidedly malignant, but extirpation is deemed impossible or unjustifiable, the rule is the same: the health must be improved and the disease as far as possible retarded. —*Mr. Druitt.*

